# Favipiravir in Therapy of Viral Infections

**DOI:** 10.3390/jcm10020273

**Published:** 2021-01-13

**Authors:** Ryta Łagocka, Violetta Dziedziejko, Patrycja Kłos, Andrzej Pawlik

**Affiliations:** 1 Department of Conservative Dentistry and Endodontics, Pomeranian Medical University, 70-204 Szczecin, Poland; lagocka@pum.edu.pl; 2 Department of Biochemistry and Medical Chemistry, Pomeranian Medical University, 70-204 Szczecin, Poland; viola@pum.edu.pl (V.D.); patison@pum.edu.pl (P.K.); 3 Department of Physiology, Pomeranian Medical University, 70-204 Szczecin, Poland

**Keywords:** favipiravir, viral infection, influenza

## Abstract

Favipiravir (FPV) is a novel antiviral drug acting as a competitive inhibitor of RNA-dependent RNA polymerase (RdRp), preventing viral transcription and replication. FPV was approved in Japan in 2014 for therapy of influenza unresponsive to standard antiviral therapies. FPV was also used in the therapy of Ebola virus disease (EVD) and severe acute respiratory syndrome coronavirus 2 (SARS-CoV-2) infection. In this review, we discuss the mechanisms of action, pharmacokinetic parameters, toxicity, and adverse effects of FPV, as well as clinical studies evaluating the use of FPV in the therapy of influenza virus (IV) infection, EVD, and SARS-CoV-2 infection, along with its effectiveness in treating other human RNA infections.

## 1. Introduction

Favipiravir (FPV) (molecular formula: C_5_H_4_FN_3_O_2_; molecular weight: 157.1 g/mol; synonyms: 6-fluoro-3-hydroxy-2-pyrazinecarboxamide; T-705; 259793-96-9; and 6-fluoro-3-oxo-3,4-dihydropyrazine-2-carboxamide) is a purine nucleoside precursor. It acts as a competitive inhibitor of RNA-dependent RNA polymerase (RdRp). FPV, originally known as T-705, was discovered and synthesized by Toyama Chemical Co., Ltd. (Tokyo, Japan). Through the screening of a chemical library of Toyama Chemical Co., Ltd., using a plaque reduction assay for antiviral activity against the influenza virus (IV), A/PR/8/34, a lead compound, which was designated as T-1105 afterward, was found to be effective. Thereafter, its derivatives were synthesized to evaluate structure-activity studies in terms of in vitro and in vivo antiviral activities, as well as pharmacokinetic properties. Then, T-705 and the related pyrazinecarboxamide compounds (T-1105 and T-1106) were selected for further investigation as drug candidates [1,2,3]. In 2014, T-705 (FPV) was approved as a drug in Japan for novel epidemic influenza strains that were unresponsive to standard antiviral therapies [3].

## 2. Mechanisms of Action

FPV is a prodrug that undergoes metabolic activation through ribosylation and phosphorylation to form the activated metabolite FPV-ribofuranosyl-5′-triphosphate (T-705RTP) in the tissues [2,4]. The mechanism of action of FPV is novel, compared to existing influenza antivirals, which inhibit the M2 ion channel of the virion (amantadine and rimantadine) or viral neuraminidase (oseltamivir, zanamivir, peramivir, and laninamivir). These drugs primarily prevent entry and exit of the virus from cells [1,2]. The primary mechanism of action of FPV binds to and inhibits RdRp, which ultimately prevents viral genome RNA transcription and replication [1,2,4]. Since RdRp domains are not present in human cells and are conserved among RNA viruses, this distinct specific mechanism targeting RNA viral polymerases makes FPV an attractive drug candidate.

In the cell, favipiravir behaves as a purine analogue and is converted into its ribofuranosyl 5′-triphosphate metabolite (T-705RTP), after which it can be incorporated in the growing RNA strand, as was with the influenza virus RNA-dependent RNA polymerase (RdRp) [1]. However, the precise molecular mechanism behind its broad-spectrum antiviral activity is yet to be fully elucidated. There are several hypotheses as to how FPV interacts with RdRp [1,2,4,5,6,7,8,9,10,11,12,13,14,15]. Some studies suggest that T-705 inhibits viral RNA synthesis through chain termination of the nascent viral RNA strand [6]. Jin et al. indicated that FPV was efficiently incorporated into the nascent RNA strand and the single incorporation of its molecule partially prevents further extension of the RNA strand. Then, the consecutive incorporation of FPV thoroughly prevents further extension of the RNA strand [5]. Other reports support the induction of lethal mutagenesis. The participation of lethal mutagenesis in the antiviral activity of favipiravir and derivatives was suggested for some virus–host systems through increase of the mutant spectrum complexity, when the virus was on its way toward extinction [7,8,9,10,11,12,13,14,15]. The study by Borrego et al. showed that the mechanism of action of favipiravir against Rift Valley fever virus (RVFV) in cell culture was due to the accumulation of mutations in the viral genome that led to a progressive decrease in viable viral progeny [13]. In this work, it was shown that favipiravir at concentrations below the toxicity threshold estimated for cells was able to extinguish RVFV from infected cell cultures. Nucleotide sequence analysis documented RVFV mutagenesis associated with virus extinction, with a significant increase in G to A and C to U transition frequencies, and a decrease of specific infectivity, hallmarks of lethal mutagenesis. Additionally, a study by Gallego et al. described the synergistic lethal mutagenesis of hepatitis C virus (HCV) through a combination of FPV and ribavirin [15].

Studies also found that the presence of purine analogues could reduce the antiviral activity of FPV, suggesting competition between this and purine nucleosides for RdRp binding. These results suggest that FPV acts as a pseudopurine [1,16]. The research conducted by Delang et al. and Abdelnabi et al. presented a possible mechanism of resistance to FPV observed in experiments with chikungunya virus (CHIKV) [17,18]. In the study by Delang et al., several favipiravir-resistant CHIKV variants were independently selected, and all acquired a unique K291R mutation in the RNA-dependent RNA polymerase (RdRp). Reverse-engineering of this K291R mutation into an infectious clone of CHIKV, confirmed the link between the mutant genotype and the resistant phenotype. It was demonstrated that the K291R mutation in the F1 motif of the RNA-dependent RNA polymerase (RdRp) of CHIKV was responsible for low-level resistance to T-705 [17]. Abdelnabi et al. reported the key role of a highly conserved lysine residue of the viral polymerase in the broad-spectrum antiviral activity of favipiravir (T-705), against positive-sense single-stranded RNA viruses. Substitutions of this conserved lysine have a major negative impact on the functionality of the RdRp. Furthermore, this lysine is involved in the fidelity of the RdRp, where the RdRp fidelity influences the sensitivity of the virus for the antiviral efficacy of FVP [18].

## 3. Drug Administration

FPV under license of the Toyama Chemical Co., Ltd. (Fujifilm Group, Tokyo, Japan) is available as Avigan^®^ (Fujifilm, Tokyo, Japan), film-coated tablets containing 200 mg of FPV [19]. The approved FPV dose for influenza in Japan is 1600 mg, twice a day on day 1, followed by 600 mg twice a day for 4 days [19,20,21]. The FPV dosing regimens used in various viral infections in humans from the best documented clinical trials are presented in Table 1. Intravenous preparations were developed to improve the oral administration of FPV.

In 2016, Fujifilm licensed FPV to Zhejiang Hisun Pharmaceutical Co. of China (Shanghai, China). It became a generic drug in 2019, allowing the company to produce it in China. In 2019, the patent of the compound of this agent expired, and it became a generic drug that other manufacturers could produce.

## 4. Absorption, Distribution, and Route of Elimination

FPV has complex, nonlinear, time, dose, and weight-dependent pharmacokinetics [3,19,32]. Phase III clinical trials showed that FPV is both metabolized by and inhibits aldehyde oxidase (AO), so an initial oral loading is required to obtain adequate blood levels [3,33]. Food has no important effect on the absorption of FPV. When FPV is given at a higher dose or in multiple doses, irreversible inhibition of AO occurs and the effect of food on the maximum drug concentration (Cmax) is lessened [3]. The bioavailability of FPV is almost complete at 97.6%. The pharmacokinetic values of FPV for the Cmax for the recommended dosing schedule is 51.5 µg/mL; the maximum drug concentration time (Tmax) is 1 h; and the half-life (T1/2) is 4.8–5.6 h [3,19,34].

Although the elimination T1/2 of FPV is estimated to range from 2 to 5.5 h (~4 h), human data on intracellular concentrations of the triphosphate in the respiratory tract are lacking [33]. Furthermore, the lower than predicted blood levels observed in patients with Ebola virus disease (EVD) and severe influenza raise concerns about its bioavailability or altered metabolism in seriously ill persons [35,36].

A phase III clinical evaluation of the drug showed that in healthy adult subjects, after single-dose administration of FPV, in the plasma and urine, the following were found—FPV, 6-fluoro-3,5-dihydroxy-2-pyrazinecarbox amide (M1), and a glucuronide conjugate of FPV (M2). There were no differences in plasma or urine metabolite species between multiple and single oral doses [3,33]. FPV was extensively metabolized with metabolites excreted mainly in the urine. The antiviral undergoes hydroxylation primarily by AO and to a lesser extent by xanthine oxidase to the inactive product M1, which is an inactive dead-end metabolite.

FPV or its metabolites were detected in semen and breast milk [3,33].

## 5. Toxicity

Based on single-dose toxicity studies, the lethal dose for oral and intravenous FPV in mice is estimated to be >2000 mg/kg. In rats, the lethal dose for oral administration is >2000 mg/kg, while the lethal dose in dogs and monkeys is >1000 mg/kg. Symptoms of overdose appear to include, but are not limited to, reduced body weight, vomiting, and decreased locomotor activity. In repeat-dose toxicity studies involving dogs, rats, and monkeys, notable findings after administration of oral FPV included adverse effects on hematopoietic tissues (such as decreased red blood cell production), increases in liver function parameters (such as aspartate aminotransferase, alkaline phosphatase, alanine aminotransferase, and total bilirubin), and increased vacuolization in hepatocytes [3].

Data from reproductive and developmental toxicity studies obtained during the drug registration process showed early embryonic deaths in rats and teratogenicity in multiple species, with exposure levels similar to or lower than those in humans [3]. Treatment with FPV might have a higher teratogenic risk compared to that in other drugs in the same class in clinical practice [3]. FPV is also associated with reversible histopathological changes in the testis and abnormal sperm in animals, but only during a prolonged treatment duration. According to these data, in Avigan^®^ labelling [19], there are warnings that it is contraindicated in women who might be or are pregnant, and in lactating women, due to its association with embryonic deaths and teratogenicity in animal studies, and that men should use the most effective contraceptive methods (including condoms) in sexual intercourse, and not have sexual intercourse with women during treatment and for 7 days afterwards. In juvenile animals, FPV causes abnormalities in musculature and mortality during prolonged dosing [3]. Toxicity information regarding FPV in humans is not readily available.

### Adverse Effects in Humans

FPV is reasonably well tolerated in clinical studies, although it is associated with dose-related, asymptomatic increases in serum uric acid levels and should be used with care in patients with gout or a history of gout, and in those with hyperuricemia [33]. Other adverse events might include mild to moderate diarrhea, asymptomatic increase of transaminases, and uncommonly decreased neutrophil counts [32]. The phase 2 randomized clinical trial involved 386 influenza patients with unspecified severity, who were administered FPV (1200 mg po b.i.d. for 1 d, followed by 800 mg po b.i.d. for 4 d) vs. placebo reported inconclusive results regarding whether FPV caused diarrhea [20]. The clinical report by Jacobs et al. showed that FVP used as post-exposure prophylaxis (PEP) in Ebola virus disease (EVD) prevention was well tolerated with no clinical adverse effects [37]. Additionally, in a study by Sissoko et al., FPV was administered in 111 patients with EVD (6000 mg on day 0, then 2400 mg daily on days 1 to 9) [24]. Among 99 adult and adolescent patients evaluated, it was generally well tolerated. Vomiting within 30 min of pill intake occurred in 30 instances (2%) in 21 patients. No severe adverse events were observed [24]. In a randomized, controlled trial by Chen et al., FVP was given to 116 patients with COVID-19 (3200 mg the first day, followed by 1200 mg/day for 10 days) [26]. Adverse effects caused by the drug were mild and manageable. The most frequently observed FPV-associated adverse event was raised serum uric acid (16/116). Additionally, in a clinical trial by Cai et al. in patients who received FPV (3200 mg the first day, followed by 1200 mg on days 2–14) to treat COVID-19, the adverse events were rare and tolerable, and none of the patients needed to discontinue FPV treatment [27]. Adverse reactions occurred in 11.4% of patients (4/35). Two patients had diarrhea, one patient had liver injury, and one patient had a poor diet. The results of the study suggest that the treatment duration of FPV might be prolonged, if necessary [27].

## 6. Influenza Viruses Infections

Since FPV was developed, many in vitro and in vivo in animal models studies confirmed that it inhibits all serotypes and strains of influenza A, B, and C viruses against which it was tested, including A(H1N1) pdm09, A(H5N1), and A(H7N9) avian virus [1,2,5,7,16,38,39,40,41,42,43,44,45,46,47,48,49,50].

## 7. Preclinical Studies

In vitro studies using laboratory strains of influenza viruses (IV) showed that FPV inhibits growth of all types (A, B and C) of IV [38,39]. It also inhibits influenza strains resistant to current antiviral drugs, which inhibit the virion M2 ion channel (amantadine) or viral neuraminidase (oseltamivir and zanamivir), and A(H5N1) and A(H7N9) avian viruses [2]. In the study by Baranovich et al., the serial passage of in vitro IV with increasing concentrations of FPV drives guanosine to adenine nucleotide mutations in IV was observed, essentially resulting in the production of non-viable viruses [7]. Additionally, an in vivo study conducted by Sidwell et al. showed that FPV protects mice against lethal infection, through a variety of IV strains [40]. The studies performed in animal models (mice) by Kiso et al., Itoh et al., and Watanabe et al. demonstrated that FPV effectively protects mice from lethal infection with oseltamivir-sensitive or oseltamivir-resistant highly pathogenic H5N1 viruses, and it had an inhibitory activity, similar to or greater than that of oseltamivir and zanamivir, against the A(H1N1)pdm09 and A(H7N9) virus [41,42,43]. FPV tested in combination with other drugs (oseltamivir and peramivir) in mice infected with A/Victoria/3/75(H3N2), A/Duck/MN/1525/81(H5N1), and A/California/04/2009(H1N1) showed synergistic improvement and 100% animal survival [44,45,46]. Several studies in mice also demonstrated that FPV administration up to 72 h after infection with seasonal IV strains, such as H1N1 and avian strains (e.g., H5N1 and H7N9), resulted in a dose-dependent reduction of lung viral titers and host mortality [2,38,40,41]. In vitro studies showed that IV presented either no or only modest reductions in susceptibility on FPV, during multiple in vitro passages [7,47,48]. The study performed by Cheung et al. found only one mutation (V43I in PB1; obtained in virus-infected cell cultures under selection) that conferred a slight increase in resistance to FPV [49]. Additionally, a study by Goldhill et al. showed a A(H1N1)pdm09 IV variant with 30-fold reduced susceptibility to FVP [50].

## 8. Clinical Studies

After many in vitro and in vivo tests in animal models, FPV was approved for clinical evaluation. During the process of drug registration and approval, multiple unpublished clinical studies with varying dose regimens were conducted in adults with acute, uncomplicated influenza [3]. A phase 3 study was completed in Japan, and two phase 2 studies were completed under a U.S. IND, with the latter funded by the U.S. Department of Defense. Descriptions of past and current trials of FPV under the U.S. program can be found on the website www.clinicaltrials.gov. One dose-ranging RCT in uncomplicated influenza (NCT01728753) found that a two times daily (BID) dosing regimen (1800 mg BID on day 1 and 800  mg BID on days 2–5) gave better antiviral and clinical effects than a three times daily (TID) dosing regimen. It also demonstrated a significantly faster time to alleviation of influenza symptoms (median, 82.3 h vs. 97.3  h) and viral load reductions, compared to the placebo group [51]. This dose regimen that was later approved to treat influenza, was tested in the next two clinical trials (NCT02008344; NCT02026349) with adults with uncomplicated influenza. Among the influenza-infected study participants, one study (*n * = 594) found a significant difference of 14.2  h in median time to alleviation of symptoms and faster reductions in nasal virus titers in the FPV recipients, compared to those receiving placebo, but the other study (*n*  = 668) found only a 6.1  h difference in time to illness alleviation [52]. Although FPV was approved in Japan, its use is restricted to occasions when the government issues a special permit, such as in the case of infection with a new influenza subtype or when a IV with reduced effectiveness to the other licensed antiviral drugs is observed [53]. It is connected with adverse reactions observed in animal model studies, such as teratogenicity and embryotoxicity, which restricted its use for the treatment of influenza in everyday practice [53,54].

The studies in seriously ill or hospitalized influenza patients are limited. Wang et al. reported comparative study of effectiveness of combined FPV and oseltamivir therapy vs. oseltamivir monotherapy in critically ill patients with IV infection [23]. A total of 40 patients were treated with combination therapy (FPV and oseltamivir), and 128 patients were treated with oseltamivir alone. Clinical improvement on day 14 in the combination group was higher than that in the monotherapy group (62.5% vs. 42.2%; *p* = 0.0247). The proportion of undetectable viral RNA at day 10 was higher in the combination group, compared to that in the oseltamivir group (67.5% vs. 21.9%; *p* < 0.01). No significant differences were observed in mortality or other outcomes. FPV and oseltamivir combination therapy might accelerate clinical recovery compared to oseltamivir monotherapy in severe influenza, and this strategy should be formally evaluated in a randomized controlled trial.

Although FPV was originally developed to treat influenza, the RdRp catalytic domain (the primary target of FPV) was expected to be similar for other RNA viruses. This conserved RdRp catalytic domain contribute to the broad-spectrum coverage of FPV. Given its efficacy at targeting several strains of influenza, it was investigated to treat novel viruses, including Ebola virus and most recently, SARs-Cov-2.

## 9. Ebola Virus Disease

The systematic literature review conducted by Lee et al. in 2019 revealed 21 anti-Ebola virus agents tested [55]. The current therapies with the best preclinical or clinical evidence for their use in EVD treatment are FPV, an RNA-polymerase inhibitor; TKM–Ebola (Tekmira Pharmaceuticals, Burnaby, BC, Canada), a combination of small interfering RNAs; and passive immunotherapy, with specific combinations of three anti-Ebola virus monoclonal antibodies (ZMapp, Mapp Biopharmaceuticals, San Diego, CA, USA, and closely related agents) [56,57]. However, data for these agents from either preclinical studies or experience in people seem to be insufficient. In the PubMed database, in August 2020, 68 results were found after entering keywords, such as FPV and Ebola virus, but among them, there was only one systematic review and two non-randomized clinical trials [24,25,55].

Two studies were performed concerning the use of FPV in Ebola virus infection in a mouse model [58,59]. The study by Smither et al. [58] showed the efficacy of FPV in post-exposure prophylaxis (PEP), and the study by Oestereich et al. [59] presented a therapeutic mouse model of EVD. Treatment with FPV from 6 to 13 days after lethal infection with Ebola virus, cured all mice when the treatment was started at the initiation of liver damage and virus detection in blood. However, later, the administration of FPV, from 8 to 14 days, prolonged the survival, but four of five mice died when the liver damage and viremia advanced. Studies showed that only early treatment with FPV was effective and treatment should be started before the liver damage progresses to irreversible levels.

On the basis of previous animal studies and FPV dose regimen extrapolation from the animal model to the human model, as well as substantial safety data from clinical trials during FVP development and licensing in Japan, Mentré et al. proposed the following dose regimen of FPV to treat humans with Ebola virus infection—6000 mg on the first day (a loading dose of 2400 mg, a second dose of 2400 mg 8 h after the first dose, and a third dose of 1200 mg 8 h after the second dose) and 2400 mg/day (a maintenance dose of 1200 mg twice a day) on days 1–9 for a total of 27,600 mg, when administered for both PEP and treatment [60]. Of note, this dose regimen was 50% greater than the one in IV treatment (Table 1). To reduce the chance of relapse in EVD, it was proposed to apply the treatment for 10 days, which corresponded to the time needed for an effective antibody response [60]. As a multi-case study, Jacobs et al. presented PEP with FPV alone or FPV with other anti-Ebola agents in healthcare workers [37]. Four of eight healthcare workers, including two with maximum risk exposures from penetrating injuries with freshly used hollow-bore needles, were administered PEP with FPV alone or FPV with other anti-Ebola agents. Although the needle stick was not confirmed to result in infection, the probability of infection was high, based on previous observations [58]. The patients received FPV for 10 days, according to a study by Mentré et al. [60]; the PEP was started 8 or 10 h after exposure (loading doses of 2400 mg, 2400 mg, and 1200 mg every 8 h on treatment day 1, followed by a maintenance dose of 1200 mg twice a day, orally). Two persons received FPV alone, one person received FVP with ZMAb on day 2 after exposure (total dose 50 mg/kg, intravenously), MIL77 on day 5 after exposure (total dose 50 mg/kg, intravenously), and one FVP with MIL77 on days 2 and 5 after exposure (total 50 mg/kg per dose, intravenously). None of the people developed EVD and PEP with FVP was considered effective, as was previously observed in PEP, using a mouse model [37,58].

The systematic review conducted by Lee et al. [55] revealed two clinical trials describing the therapeutic effect of FPV in patients with West African EVD [24,25]. A non-randomized single-arm study was conducted in Guinea [24]. It included 126 patients who were administered FPV, and 111 who were analyzed and compared with 540 patients, as a historical control group. FPV was orally administered according to the recommendations by Mentré et al. [60]. FPV treatment reduced the mortality rate in the low viral load group (to 33% compared with the historical control group that was not treated with FPV), but this reduction in the mortality rate was not statistically significant. However, the study had a lack of a concurrent control group and uncertainty in patient selection criteria led to a moderate risk of bias.

The second non-randomized single-arm study was conducted by Bai et al. [25]. During the 2014 epidemic of Ebola virus infection in West Africa, patients who were treated without FPV in the period before preparation for FPV treatment were classified as historical controls, and the therapeutic efficacy of FPV was compared between the patient group treated with the drug and the historical patient group in the two clinical trials described above. The study by Bai et al. included 39 FPV-treated patients and 85 historical control patients. FPV was administered orally, 800 mg twice on day 0, and two doses of 600 mg each on subsequent days, ranging from 3 to 11 days, until discharge, transfer, or death (*n* = 39). The mortality among patients studied was 44% (17/39) in the FPV group and 65% (55/85) in the historical control group. The overall survival rate in the FPV treatment group was higher than that in the control group (56.4% (22/39) vs. 35.3% (30/85); *p* = 0.027) [25]. Both of these studies used non-random assignment of intervention, and lacked a concurrent control group, and the potential for differential between-group treatments led to a moderate risk of bias. Despite an ethical problem with the placebo group in clinical trials, randomized placebo-controlled trials are desirable for confirming therapeutic effects of FVP in Ebola virus infection.

## 10. SARS-CoV-2 Infection

With the emergence and catastrophic, rapid spread of a new pathogen, SARS-CoV-2, resulted in more than 3 million cases and more than 210,000 deaths worldwide as of April 2020, which increased to 83,715,617 confirmed cases and 1,835,901 deaths by 4 January 2012. Many clinical trials of potential COVID-19 treatments are underway [61]. Clinicians have administered a number of antiviral treatments to patients with COVID-19. One of them is FPV. Although there are a lot of results in the databases on FPV and SARS-CoV-2 studies, those with well documented efficacy and safety of antiviral treatment for COVID-19 are few.

The systemic review and meta-analysis conducted by Wei Liu et al. revealed one randomized clinical trial with FPV used in COVID-19 patients [62]. This randomized clinical trial presented by Chen et al. [26] enrolled 240 COVID-19 patients (15 persons with mixed-severity illness and the rest of the patients were non-severe (88.6%)): 120 patients received FPV (among them, 116 were assessed), and 120 received umifenovir. FPV was dosed at 3200 mg on the first day, followed by 1200 mg/day for 10 days. Among patients with COVID-19, FPV as compared to umifenovir, did not significantly improve the clinical recovery rate at day 7 (FPV group (71/116) vs. umifenovir group (62/120)). FPV significantly improved the latency to relief for pyrexia and cough. However, the study, according to the criteria of systemic review applied by Liu et al., presented very low-quality evidence [62].

A non-randomized interventional study that enrolled 80 patients with laboratory-confirmed COVID-19 was presented by Cai et al. [27]. In the trial, the medical status of 35 patients treated with FPV was compared (day 1: 1600 mg twice daily; days 2–14: 600 mg twice daily) plus interferon (IFN)-α by aerosol inhalation twice daily)), vs. 45 patients who received lopinavir (LPV)/ritonavir (RTV) (days 1–14: 400 mg/100 mg twice daily) plus IFN-α, through aerosol inhalation, twice daily. The study showed a shorter viral clearance time and significant improvement in chest imaging in patients receiving FPV, in comparison with those in patients receiving LPV/RTV. However, the study, according to the criteria of systemic review applied by Liu et al., also presents very low-quality evidence [62].

To assess the efficacy and safety of favipiravir in adults with mild-to-moderate coronavirus disease, Udwadia et al. [28] conducted a randomized, comparative, open-labeled, phase 3 clinical trial. This study included 150 adults (18–75 years) with RT-PCR confirmed COVID-19 and mild-to-moderate symptoms (including asymptomatic) (Table 1), plus standard supportive care (*n* = 75) versus supportive care alone (*n* = 75). Median time to the cessation of viral shedding was 5 days (95% CI: 4 days, 7 days) versus 7 days (95% CI: 5 days, 8 days), *p* = 0.129, and median time to clinical cure was 3 days (95% CI: 3 days, 4 days) versus 5 days (95% CI: 4 days, 6 days), *p* = 0.03, for FPV and control, respectively. Adverse events were observed in 36% of favipiravir and 8% of control patients. One control patient died due to worsening disease. Significant improvement in time to clinical cure suggests that favipiravir might be beneficial in mild-to-moderate COVID-19.

Doi et al. [29] conducted a prospective, randomized, open-label, multicenter trial of favipiravir for the treatment of COVID-19 at 25 hospitals across Japan. Eligible patients were adolescents and adults admitted with COVID-19, who were asymptomatic or mildly ill. Eighty-nine patients were enrolled in the study, 69 of whom underwent virological evaluation. Patients were randomly assigned at a 1:1 ratio to early or late favipiravir therapy. FPV administration did not significantly improve viral clearance during the first 6 days, but there was a trend towards earlier virus clearance after drug administration. None of the patients experienced progression of disease or death. Although limited by the small sample size, the results suggest an antiviral effect of favipiravir in this patient population.

Lou et al. [30] conducted an exploratory trial with 3 arms involving hospitalized adult patients with COVID-19. Thirty patients were randomly assigned in a 1:1:1 ratio into the baloxavir marboxil group, the favipiravir group, and the control group. The FPV dosage is presented in Table 1. The percentage of patients who turned viral negative after a 14-day treatment was 70%, 77%, and 100% in the baloxavir marboxil, favipiravir, and control group, respectively.

Çalik BaŞaran et al. [63] presented the results of prospective, observational, single center study, including 174 consecutive COVID-19 adult patients. The patients were treated with hydroxychloroquine (HQ) alone (13.2%), with 22 HQ plus azithromycin (AZ) (64.9%), and with regimens including favipiravir (18.4%). The length of hospital stay and time to symptom relief was longer in the FAV group, but similar in the HQ and HQ plus AZ groups.

In contrast to the results of FPV effectiveness presented by Lou et al. [30] and Çalik BaŞaran et al. [63], was the study by Ivashchenko et al. [31]. In this study, 60 patients hospitalized with COVID-19 moderate pneumonia were assessed and randomized, as presented in Table 1. Both FPV dosing regimens demonstrated similar virological response. On Day 5, the viral clearance was achieved in 25/40 (62.5%) patients on FPV and in 6/20 (30.0%) patients on standard of care (*p* = 0.018). By Day 10, viral clearance was achieved in 37/40 (92.5%) FPV patients and in 16/20 (80.0%) standard of care patients (*p* = 0.155). Adverse drug reactions (mild to moderate) was reported in 7/40 (17.5%) and caused early discontinuation of the study drug in 2/40 (5.0%) patients. Two patients on FPV 1600/600 mg were moved to the intensive care unit, received mechanical ventilation, and later died. The proportion of patients who achieved negative PCR on Day 5 on both dosing FPV regimens was twice as high, as in the control group (*p* < 0.05). In the study, FVP demonstrated rapid antiviral response against SARS-CoV-2.

Rattanaumpawan et al. [64] presented a retrospective observational study of 274 COVID-19 patients hospitalized at five hospitals in Thailand, of which 63 patients (23.0%) received favipiravir. Within two days of initiating FPV treatment, nearly all patients were prescribed a chloroquine-based agent (98.4%) and a protease inhibitor (96.8%); half of them also received azithromycin (49.2%). Only a few patients received a steroid (12.7%) or tocilizumab (6.4%) at this time. The study reports the promising effectiveness of favipiravir for treating COVID-19 patients, however, it had some limitations, because the majority of patients received a chloroquine-based agent and protease inhibitors. Therefore, the good treatment response among them might be the synergistic results of a triple combination of favipiravir, chloroquine-based agent, and protease inhibitors.

Despite the fact that the most of studies present very low-quality evidence, related to the small size of groups, the use of additional drugs, or incomplete assessment of the initial parameters of patients, the systematic review and meta-analysis carried out by Shrestha et al. [65] showed a significant clinical and radiological improvement, following treatment with FVP in comparison to the standard of care, with no significant differences on viral clearance, oxygen support requirement, and side-effect profiles.

## 11. Effectiveness of FPV in Other Human RNA Infections

There were also attempts to use FPV as an emergency or compassionate drug in treatment of diseases with high mortality, caused by other RNA viruses for which there is no effective drug (i.e., Lassa fever, norovirus, severe fever with thrombocytopenia syndrome or rabies) [66,67,68,69]. Based on the earlier reports on FPV efficacy in the treatment of lethal Lassa virus infection in animals (decreased levels of viremia and increased survival) [70,71], Raabe et al. applied FPV and ribavirin in two patients with Lassa fever [66]. Both patients survived but developed transaminitis and had prolonged detectable virus RNA in blood and semen. Ruis et al. presented the case of treatment with FPV for severe chronic norovirus infection in a 48-year-old man with common variable immunodeficiency. Despite the symptomatic response to treatment with FPV, the patient died [67].

FPV was also tested as a drug in severe fever with thrombocytopenia syndrome (SFTS), which is an emerging tick-borne infectious disease caused by the novel bunyavirus-SFTS virus (SFTSV) [68,72].

Several studies reported favipiravir to be effective in treating of other viral diseases modeled in vitro and in rodent systems [13,73,74,75,76,77]. Julander et al. [73] studied the favipiravir effectiveness in Yellow fever virus (YFV)-infected hamsters. They achieved significant improvement in disease parameters after FPV treatment. Notably, the related pyrazine derivative, T-1106, was found to be more effective than T-705, in treating yellow fever virus infection. In the study by Borrego et al. [13], treatment with favipiravir was shown to reduce the infectivity of Rift Valley fever virus, both in cell cultures and in experimental animal models. FPV was also studied in treating hepatotropic Punta Toro virus (PTV, Phlebovirus) infection in rodents with promising results [74]. The study of Safronetz et al. [75] evaluated the efficacy of favipiravir against the Sin Nombre virus (SNV) and Andes virus (ANDV), the predominant causes of Hantavirus pulmonary syndrome (HPS) in North and South America, respectively. Oral T-705 therapy remained protective against HPS when treatment was initiated prior to the onset of viremia in hamster model. Based on these findings, the authors suggest that T-705 treatment is beneficial for post-exposure prophylaxis against HPS-causing viruses and should be considered for probable exposures in humans. Oestereich et al. [76] showed that favipiravir is highly potent against Crimean-Congo hemorrhagic fever (CCHF) virus, in in vitro and in vivo mice model. Its in vivo efficacy was superior to that of the current standard drug for the treatment of CCHF, ribavirin. Yamada et al. [77] studied the efficacy of favipiravir in rabies, post-exposure prophylaxis. Rabies is the fatal encephalitis caused by rabies virus (RABV), and no antiviral drugs for RABV are currently available. The authors reported the efficacy of favipiravir against RABV in vitro and in vivo mice model. The results suggest that FPV is active against RABV and might serve as a potential alternative to rabies immunoglobulin in rabies post-exposure prophylaxis.

The most valuable clinical studies examining the role of FPV in various viral infections in humans are presented in Table 2.

## 12. Conclusions

FPV as a drug shows significant antiviral efficacy in in vitro studies and in vivo animal studies. Despite many publications presenting FPV as a drug with high effectiveness in human viral infections, many are non-randomized trials with low-quality evidence, case reports, letters to redactions, or non-scientific journalistic reports. As a result, there is a need to confirm the effectiveness of FPV in viral infections as a multicenter, randomized, double-blind clinical trial on a large group of patients. Additionally, further studies at higher doses, in combination with other antivirals are needed to determine the safety and efficacy of FPV in high-risk and seriously ill patients.

## Figures and Tables

**Table 1:** Favipiravir (FPV) dosing regimens used in various viral infections in humans presented in the best documented clinical trials.

Type of Viral Diseases	FPV Dosage	References
Treatment of uncomplicated influenza Studies before drug registration	Low-dose regimen: Day 1: 2000 mg (1000 mg twice a day) Day 2–5: 800 mg (400 mg twice a day) High-dose regimen: Day 1: 2400 mg (1200 mg twice a day) Day 2–5: 1600 mg (800 mg twice a day)	[20]
	Day 1: 3600 mg (1800 mg twice a day) Day 2–5: 1600 mg (800 mg twice a day)	[21]
	Day 1: 3600 mg (1800 mg twice a day) Day 2–5: 1600 mg (800 mg twice a day)	[22]
The approved FPV dose for influenza in Japan	Day 1: 3200 mg (1600 mg twice a day) Day 2–5: 1200 mg (600 mg twice a day)	[19]
Treatment of severe influenza patients (combination therapy FPV and oseltamivir)	Day 1: 3200 mg (1600 mg twice a day) Day 2–5: 1200 mg (600 mg twice a day)	[23]
Treatment of Ebola virus disease (EVD)	Adults: Day 1: 6000 mg ((first dose: 2400 mg; second dose (8 h after the first dose): 2400 mg; third dose (8 h after the second dose) 1200 mg. Day 2–10: 2400 mg (1200 mg twice a day) Children: The dose was adapted according to body weight	[24]
	Day 1: 1600 mg (800 mg twice a day) Subsequent days, ranging from 3 to 11 days: 1200 mg (600 mg twice a day) until discharge, transfer, or death	[25]
Treatment of COVID-19 patients	Day 1: 3200 mg (1600 mg twice a day) Day 2–10: 1200 mg (600 mg twice a day)	[26]
	Day 1: 3200 mg (1600 mg twice a day) Day 2–14: 1200 mg (600 mg twice a day) plus interferon (IFN)-α by aerosol inhalation twice daily	[27]
	Day 1: 3600 mg (1800 mg twice a day) Day 2–14: 1600 mg (800 mg twice a day) plus standard supportive care	[28]
	Day 1: 3600 mg (1800 mg twice a day) Day 2 for a total of up to 19 doses over 10 days; 1600 mg (800 mg twice a day)	[29]
	The first dose was 1600 mg or 2200 mg orally, followed by 600 mg each time, three times a day, and the duration of administration was not more than 14 days.	[30]
	Day 1: 3200 mg (1600 mg twice a day) Day 2–14: 1200 mg (600 mg twice a day) or Day 1: 3600 mg (1800 mg twice a day) Day 2–14: 1600 mg (800 mg twice a day)	[31]

**Table 2:** The most valuable clinical studies examining the role of favipiravir (FVP) in various viral infections in humans.

Type of Virus	Type of Study	Study Groups	The Most Important Efficacy Results	References
**IV**	Comparative study of two separate prospective trials	40 patients with severe influenza treated with combination therapy FPV and oseltamivir vs. 128 patients with severe influenza treated with oseltamivir monotherapy	Clinical improvement on day 14 in the combination group was higher than that in the monotherapy group (62.5% vs. 42.2%; *p* = 0.0247). No significant differences were observed in mortality.	[23]
**EVD**	Historically controlled, single-arm proof-of-concept trial	Group of 126 patients, of whom 111 were analyzed: adults and adolescents, ≥13 yo, *n* = 99; young children, ≤6 yo, *n* = 12 vs. 540 patients as a historical control group	Study provided no evidence that FVP monotherapy at this dose might have a favorable benefit/risk ratio in patients with very high viral load. FVP treatment reduced the mortality rate in the low viral load group but reduction in the mortality rate was not statistically significant.	[24]
	Retrospective clinical case series study	39 FVP-treated patients vs. 85 historical control patients	The overall survival rate in the FVP treatment group was higher than that in the control group (56.4% (22/39) vs. 35.3% (30/85); *p*= 0.027). The average survival time of the treatment group (46.9 ± 5.6 days) was longer than that of the control group (28.9 ± 4.7 days).	[25]
**SARS-CoV-2**	Prospective, randomized, controlled, open-label multicenter trial	240 COVID-19 patients (15 persons with mixed-severity illness and the rest of patients were non-severe (88.6%)): 120 FVP-treated patients (among them, 116 were assessed) vs. 120 umifenovir-treated patients	FVP, compared to umifenovir, did not significantly improve the clinically recovery rate at day 7.	[26]
	Open-label before-after controlled trial	35 FVP-treated patients vs. 45 patients who have received lopinavir/ritonavir	FPV showed better treatment outcomes in terms of disease progression and viral clearance.	[27]
	Randomized, comparative, open-labeled, phase 3 clinical trial.	75 adults (18–75 years) with RT-PCR confirmed COVID-19 and mild-to-moderate symptoms (including asymptomatic) FVP treated plus standard supportive care vs. 75 patients with supportive care alone	Significant improvement in time to clinical cure suggests favipiravir may be beneficial in mild-to-moderate COVID-19	[28]
	Prospective, randomized, open-label, multicenter trial	89 asymptomatic or mildly ill patients: 44 FPV early treatment group vs. 45 FPV late treatment group	While limited by the small sample size, the findings suggest antiviral activity of favipiravir in this patient population.	[29]
	Exploratory trial	10 baloxavir marboxil-treated patients vs. 10 FVP-treated patients vs. control group (*n* = 10)	The study findings could not prove a benefit of addition of either baloxavir marboxil or favipiravir under the trial dosages to the existing standard treatment.	[30]
	Randomized trial	60 patients with COVID-19 moderate pneumonia: 20 FVP-treated patients (lower dose) vs. 20 FVP-treated patients (higher dose) vs. 20 patients with supportive care	The proportion of patients who achieved negative PCR on Day 5 on both dosing regimens of AVIFAVIR was twice as high as in the control group (*p* < 0.05).	[31]
